# The Impact of DNA Methylation on IL6 mRNA Levels in Hematinic Deficiency and Atopy-Associated Recurrent Aphthous Stomatitis Patients

**DOI:** 10.1155/2021/5560695

**Published:** 2021-04-12

**Authors:** Nanan Nur'aeny, Dida Akhmad Gurnida, Oki Suwarsa, Irna Sufiawati

**Affiliations:** ^1^Faculty of Medicine, Universitas Padjadjaran, Bandung, Indonesia; ^2^Child Health Department, Faculty of Medicine, Universitas Padjadjaran, Bandung, Indonesia; ^3^Dermatology and Venereal Disease Department, Faculty of Medicine, Universitas Padjadjaran, Bandung, Indonesia; ^4^Oral Medicine Department, Faculty of Dentistry, Universitas Padjadjaran, Bandung, Indonesia

## Abstract

**Objective:**

To investigate the DNA methylation using pyrosequencing and its effects on the upregulation of IL6 mRNA in patients with recurrent aphthous stomatitis (RAS) in connection with hematinic deficiency and atopy. *Material and Methods*. This cross-sectional study was conducted at Dr. Hasan Sadikin Hospital, Bandung, from January–March 2019 and was approved by the Health Research Ethics Committee of Universitas Padjadjaran (Ethics No. 990/UN6.KEP/EC/2018). Furthermore, the subjects had RAS ulcers with a history of at least twice a year along with atopy and dietary imbalance with no history of recurrent intraoral herpes or any systemic diseases. This study was performed on 23 RAS patients and 21 healthy subjects, and the sampling was carried out consecutively. The blood samples were collected from all the subjects, and then, the DNA and RNA were extracted from the peripheral blood mononuclear cells (PBMCs). Consequently, the bisulfite-modified DNA was used to confirm the methylation status of the IL6 gene promoter through the pyrosequencing method. The methylation levels of the IL6 promoter were assessed by a reverse transcriptase-polymerase chain reaction technique. The gene expression of RAS and the control group was analyzed by the 2^−ΔΔ^*C*_T_ method. The statistical analysis using the Mann–Whitney *U* test was conducted to evaluate IL6 mRNA levels and DNA methylation with *p* value <0.05 considered to be statistically significant.

**Result:**

The IL6 mRNA levels were approximately 1.88-fold in RAS patients, and there was a significant relationship between the expression of the IL6 gene and the increased risk of RAS (*p* < 0.001). It was reported that four out of six sites in the cytosine phosphate guanine (CpG) island IL6 promoter had a lower degree of methylation, and two other sites in patients with RAS had greater methylation compared with control, but not statistically significant.

**Conclusion:**

This study showed the upregulation of IL6 mRNA levels in RAS patients compared to control. DNA methylation in the present study is at sites 566–658, whereas the location of the IL6 promoter is at sites 1–1684. Thus, it would be necessary conducting some research at other CpG sites of IL6 promoter islands to determine the status of DNA methylation.

## 1. Introduction

Recurrent aphthous stomatitis (RAS) is a common oral ulcer discovered by dental professionals worldwide, and the prevalence of RAS varies between populations. Furthermore, the prevalence of RAS was 55% and 35.6% discovered in the dental clinic as of 2010–2012 and 2014-2015, respectively [1, 2]. Other studies carried out in the midst of the population of 18-year-old adolescents in Brazil discovered to be 24.9% [3], about 10.84% RAS were also discovered in Turkey [4], 14% was reported in the female dental students in King Khalid University Saudi Arabia [5], and 29.38% was reported in the college students in Beijing University of Chinese Medicine [6]. RAS is considered as a multifactorial process of unknown etiology, in which various triggering factors and an immunological disturbance are combined, including genetic [7, 8], vitamin B-12, folate, and iron deficiency [9, 10] and an allergic condition, such as atopy [11, 12] Pain or unpleasant feeling and its recurrency in the oral cavity are always complained by an individual that has RAS, and this condition makes researchers interested in discovering the pathogenesis of RAS and its ultimate goal in finding the appropriate novel therapy [13].

RAS is a chronic oral inflammatory disease with an inflammatory mechanism, and one of the essential modulators that may induce and determine the type of immune response is interleukin 6 (IL6) [14]. The immune system and aberrant cytokine cascade are believed to be critical in the episode of RAS ulcers [15, 16]. The previous studies on the immunological profiles of the RAS patients showed a significant difference in the distribution of the serum cytokines levels, mainly interleukin-6 (IL6), known as the most potent proinflammatory cytokines in which about 16% is discovered in RAS and 0% in control (), and this is shown to be significantly higher in the RAS group than in control [17].

Atopy is the genetic predisposition to develop allergic symptoms triggered by environmental antigens (allergens), potentiated by an underlying aberrant Type 2 inflammatory process [18, 19]. Atopy is a disease that has an atopic march, ranging from mild to severe symptoms. Individuals with a family history of atopy have a high tendency to experience the aforementioned conditions, such as allergic rhinitis, atopic dermatitis, and even bronchial asthma [20]. The immune response in atopy is characterized by the secretion of IL-4, IL-5, and IL-13. However, IL6 has been shown to promote the differentiation of CD4+ T cells into Th2 cells that produce IL-4. A previous study found that IL6 levels were specifically elevated in asthmatic subjects compared with healthy controls [21]. Some studies showed that higher associations between polymorphism at IL6−174 G/C (rs 1800795) and bronchial asthma were observed in atopic patients [22]. Gharagozlou et al. also performed a study and found that the G allele was significantly more frequent at position -174 in IL6 in atopic patients than in the healthy controls [23]. Based on evidence from many studies, IL6 in atopy is one of the main inflammatory mediators which also plays a major role in the recurrence of RAS. A preliminary study also showed a significant difference in IL6 log levels in the atopic and RAS group compared with the control group [24].

The IL6 gene encodes a cytokine that functions in inflammation and the maturation of B cells [25]. The protein is primarily produced at sites of acute and chronic inflammation, where it is secreted into the serum [26]. The role of genetic variability of the IL6 gene (rs1800795) has been examined in RAS [27–29]. RAS is known to have a genetic background, as atopy and both diseases could also be influenced by environmental factors due to some lifestyle factors such as consumption of food with unbalanced nutrition [30–32].

Epigenetics is recognized as one of the most crucial mechanisms in regulating gene function and expression for different cell types [33]. Nutrients and other bioactive food components potentially regulate gene expressions, such as folate and vitamin B-12. Folate is a metabolic cofactor by carrying and chemically activating single carbons for the biosynthesis of purine nucleotides and thymidylate and for the remethylation of homocysteine to methionine, a metabolic network known as folate-mediated one-carbon metabolism. Methionine, in turn, can be adenosylated to form S-adenosylmethionine (SAM), which is a cosubstrate for several cellular methylation reactions. Purines and thymidylate are required for DNA synthesis, and SAM is required for genome methylation [34].

**Figure 1:**
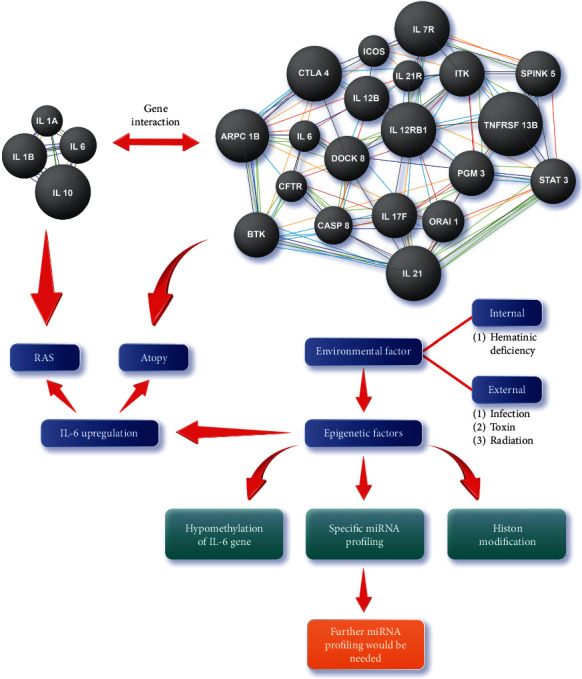
Overview of the genomic and epigenomic analysis of the three related conditions: recurrent aphthous stomatitis (RAS), atopy (such as rhinitis allergy, bronchial asthma, and dermatitis atopy), and hematinic deficiency. The grey balls are genes responsible for RAS (left side) and atopy (right side). They interact by coexpression, sharing protein domain, and even direct gene interaction [37]. IL6 is the most important proinflammatory gene affecting RAS and atopy, while its upregulation is interfered with by some external factors (infection, toxin, or radiation), internal factors (folic acid, iron, or B-12 deficiency), as an environmental factor. Also, some epigenetic factors interfere with IL6 gene expression. Strong evidence support hematinic deficiency as a crucial internal environmental factor that affects DNA methylation of some genes. There is no previous study that analyzes the DNA methylation of some important genes in RAS, notably linking with the history of atopy.

DNA methylation is a form of epigenetic factor that is closely related to macronutrients and micronutrients. Individuals who are malnourished usually also have micronutrient deficiency, including hematinic [35]. An impaired DNA methylation state can be resolved by appropriate dietary intervention [36]. RAS therapy that has been commonly used is multivitamins supplementation, and most people are not familiar with folic acid, iron, or vitamin B-12 for RAS therapy. This present study is the first study to determine whether DNA methylation plays an essential role in the expression of the IL6 gene in individuals who have RAS with a history of atopy and hematinic deficiency (Figure 1).

## 2. Materials and Methods

### 2.1. Patients and Healthy Control Study Group

The cross-sectional study was conducted at Dr. Hasan Sadikin Hospital, Bandung, from January–March 2019, after approval from the Health Research Ethics Committee of Universitas Padjadjaran (Ethics No. 990/UN6.KEP/EC/2018). The target population was subjects having RAS, atopy, and hematinic deficiency. The inclusion criteria include subjects between 18 and 40 years experiencing the recurrence of RAS twice yearly. Having RAS in the active phase, with a history of atopy (atopic dermatitis/bronchial asthma/allergic rhinitis), as well as an imbalanced diet and the hematinic deficiency (iron/vitamin B12/folic acid), is based on the results of the laboratory examination (Folic acid and vitamin B-12 measured by ELISA using Rayto®, ferritin, and total IgE measured by CLIA using XPT®.) The exclusion criteria were recurrent intraoral herpes, gastrointestinal disorders, blood, and systemic diseases. The minimal sample size was 20 for each group, which arrived from an unpaired numeric analytic design. The subject recruitment was carried out based on inclusion and exclusion criteria consecutively without blinding.

Informed consent was made prior to the enrollment, and the subject was asked to fill out some questionnaire forms regarding the history of RAS, atopy, and dietary habit with the food frequency questionnaire form. An oral examination was carried out, and two forms were recorded, i.e., the odontogram form and the oral mucosa form.

The subject was prepared for 5 mL of the blood sample with the venipuncture method by a clinical pathology assistant. Complete blood count and serology (IgE, anti-HSV 1 IgM and IgG, folic acid, vitamin B-12, and ferritin) were performed. Any subjects with positive anti-HSV1 results were excluded.

### 2.2. RNA Isolation

Total RNA was extracted from peripheral blood mononuclear cells (PBMCs) according to the Zymo Research® Quick-RNATM MiniPrep Plus kit instructions catalog number R1058. The purity and concentration of the total RNA were estimated by using the Thermo Scientific Nanodrop 2000*®*, and at 260–280 nm, and the purity of RNA was assessed. The total RNA was shown by gel electrophoresis of the individual samples on a 1% agarose gel [38]. The next step after obtaining the RNA is to perform the reverse transcriptase-polymerase chain reaction (RT PCR) procedure [39].

### 2.3. RT PCR Procedure

The materials used for the RT PCR process were 10 *µ*l Sybr sensitivity, primer forward for the IL6 gene (5′-GCT TCT GAA CCA GCT TGA CC-3′) and primer reverse for the IL6 gene (5′-GCC TCA GAC ATC TCC AGT CC-3′), primer forward for gene reference (GADPH) (5′–CCC CAC ACA CAT GCA CTT ACC-3′) and primer reverse of GADPH (5′-CCT AGT CCC AGG GCT TTG ATT-3′), 0.8 *µ*l each, 0.1 *µ*l reverse transcriptase, 0.2 *µ*l ribosafe inhibitor, and 6.1 ul H_2_O. All materials were multiplied according to the number of samples examined [40]. All materials were placed into the Eppendorf tubes in a frozen state. The mixture was placed in an 18 *µ*l PCR tube, and then, a 2 ul RNA template was added in such a way that the final volume was 20 ul. The next step was to insert the PCR tubes into the RT Gene Rotor Q® PCR machine for 2.5 hours, which consists of several stages, namely, cDNA synthesis, reverse transcriptase inactivation, denaturation, and annealing. Hold cycles: 42°C, 5 minutes, hold 2 : 95°C for 2 minutes, cycling 95°C for 10 seconds, and temperature 60°C for 30 seconds for 40 times.

### 2.4. RT PCR Data Analysis

The results and data are presented as cycle threshold (*C*_T_) and then analyzed with a relative quantification method using the 2^−ΔΔ^*C*_T_ formula (Livak) [41]. The Delta *C*_T_ (Δ*C*_T_) RAS group is defined as a subtraction from the *C*_T_ IL6 and *C*_T_ gene reference (GADPH). The Delta *C*_T_ (Δ*C*_T_) control group is also calculated using the same formula. Delta Delta *C*_T_ (ΔΔ*C*_T_) is defined as subtraction between both of the Δ*C*_T_, and the gene expression achieved from the ΔΔ*C*_T_ quadrat [41, 42].

### 2.5. Preparation of DNA and Bisulfite-Treated Genomic DNA Samples

The protocol for DNA extraction from leukocytes (peripheral blood mononuclear cells/PBMC) requires tools such as centrifuges, water baths, micropipettes of various sizes (10, 200, and 1000 *µ*l), concentrators, and 1.5 ml microtubes, as well as materials including RBC lysis solution 1x, ammonium acetate 5 M, cell lysis solution, RNAse 4 mg/ml, isopropanol, 70% ethanol, and TE buffer pH 8. The DNA preparations required 300 *µ*l blood (whole blood), and 900 *µ*l 1x RBC lysis solution is added into the Eppendorf tube and then shaken several times. The solution is stored for at least 10 minutes at room temperature. After 10 minutes, the solution is placed into a centrifuge with a speed of 13000–16000 RPM for 20 seconds to obtain a leukocyte pellet (PBMC). The supernatant is removed and then repeated until there are no more red blood cells, then mixed until homogeneous by vortex for approximately 20 seconds, and 300 L of cell lysis solution is added until homogeneous by pipette to lyse the cells. RNAse 1.5 *µ*l with a concentration of 5 mg/ml was added in a tube and incubated in a 37°C water bath for at least 15 minutes.

The next step is the addition of a protein solution of precipitation (to precipitate the protein), that is, a 5 *µ*l ammonium acetate solution, which is then vortexed until the solution looks like milk and then centrifuged for 3 minutes. The supernatant containing DNA is transferred to a new Eppendorf tube filled with 600 *µ*l isopropanol, which is mixed several times until the DNA looks like a white thread. After centrifuging for 1 minute, the DNA will look like a white sediment. The supernatant is removed, and then, about 600 *µ*l of 70% cold alcohol is added. The tube is turned over several times to wash DNA. Similarly, the DNA pellets are obtained after centrifugation for 5 minutes. The alcohol is then discarded, and the tube is allowed to dry by turning it over the tissue until the alcohol evaporates. The pellet was then dissolved with 50 *µ*l TE buffer pH 8. The bisulfite-converted protocol was carried out using the Zymo EZ DNA Methylation-Gold kit® catalog D5005 [43, 44].

### 2.6. PCR Procedure

The next step after obtaining DNA preparations is to carry out the PCR procedure, with materials used in the form of Pyromark PCR master mix 12.5 *µ*l, Coralload concentrate 2.5 *µ*l, primer forward (5′-AGG GAT AAT TTA GTT TAG AGT TTA TTT GT-3′) 1 *µ*l, primer reverse (5′-CTC CCT CTC CCT ATA AAT CTT AAT T-3′) 1 *µ*l (Biotin), Q solution 5 *µ*l, Q solution 5 *µ*l, ddH2O 1 *µ*l, and DNA bisulfite *µ*l [43].

### 2.7. Pyrosequencing

The materials used for processing each sample (on plate 1) are streptavidin sepharose three *μ*l, binding buffer 37 *μ*l, PCR product 15 *μ*l, and Miliq 25 *μ*l. Plate 2 for pyrosequencing contained 40 ul annealing buffers, which has been added to the sequencing primer in such a way that the final primary concentration of each hole was 0.3 mM. Before entering the Pyromark Q96ID, the plates are first processed at the vacuum workstation, where double-stranded DNA is converted into single-stranded DNA, and the order of the primary sequence is determined. The results from plate 2 of the vacuum workstation are stored in the PyroMark Q96ID for further processing by the pyrosequencing procedure of the CpG assay to see the percent methylation.

The methylation status of the IL6 gene promoter was confirmed using the pyrosequencing method. The bisulfite-modified DNA was amplified using forward and reverse primers via PCR, allowing the conversion of PCR products into single-stranded DNA as a template suitable for pyrosequencing. All samples were heated to 95°C for 5 minutes and then amplified for 45 cycles of 95°C 45 seconds, 54°C 45 seconds, and 72°C 45 seconds, followed by the final extension at 72°C for 5 minutes. The quality and lack of PCR product contamination were examined on 2% agarose gel by staining ethidium bromide. After purifying the PCR product using Sepharose beads on the PyroMark Vacuum Preparation Workstation (Qiagen), pyrosequencing was carried out according to the manufacturer's specifications with sequencing primers (5′-ATA AGA TTT AGA TTG TGG GTG T-3) using the PyroMark Q96MD (Qiagen) System. The average methylation index (MI) was calculated from the average percentage of methylation for all observed CpG sites [43].

### 2.8. Statistical Analysis

The statistical analysis was carried out using SPSS software version 21.0 (SPSS, Chicago, IL, USA). Furthermore, the normal distribution test was performed using the Kolmogorov–Smirnov test with Lielliefors correction. The quantitative data of DNA methylation were presented as median and minimum-maximum. The gene expression data obtained were evaluated using relative quantification by the 2^−ΔΔ*C*^_T_ method [41]. Similarly, the differences between mRNA IL6 and DNA methylation between RAS and control groups were evaluated using the Mann–Whitney *U* test. A *p* value <0.05 was considered a significant difference.

## 3. Results

### 3.1. Subject Characteristics

The population consists of 81 subjects. Of them, 37 were excluded due to reactive results of HSV-1 infection. There are twenty-three RAS subjects (10 men, 13 women, age range 19–38 years old, mean age 23,5 ± 4,2 years), and the control group comprises 21 non-RAS subjects proportionally matched by gender and age (6 men, 15 women, age range 20–37 years old, mean age 22,7 ± 3,8 years). There was no significant difference in the age between RAS patients and controls (*p* > 0.05). Consequently, it was reported that the hematinic level was a significant difference between the two groups for vitamin B-12 levels (*p*=0.001) based on the Mann–Whitney test, and the atopic disease was discovered only in the RAS group (*p*=0.001) (Table 1). The dietary recall was conducted by the food frequency questionnaire (FFQ [45], where the resulted majority (91.3%) of the subjects consumed eggs and milk daily, while a few of them (8.69%) had the habit of eating fresh vegetables and fruits).

### 3.2. Reverse Transcriptase PCR for IL6 Gene

**Figure 2:**
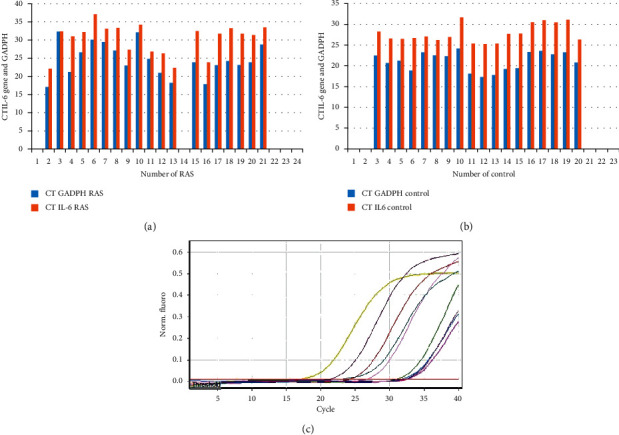
(a) The cycle threshold (*C*_T_) of the IL6 gene in the RAS group. (b) The *C*_T_ of the IL6 gene in the control group (c) Quantitation data for Cycling A.Green. The result and data from RT PCR are presented as cycle threshold (*C*_T_), and (Figures 1–3) the *C*_T_ is the cycle in which the fluorescence level reaches a specified amount (the threshold). This method (2^−ΔΔ^*C*_T_ formula) [41] immediately uses the *C*_T_ information, and the end result is normalized as a change in the expression of the target gene (IL6) in the target sample (RAS) compared to a shown reference sample (control) to a reference gene (GADPH).

The result and data from RT PCR are presented as cycle threshold (*C*_T_), and (Figure 2) the *C*_T_ is the cycle in which the fluorescence level reaches a specified amount (the threshold). This method (2^−ΔΔ^*C*_T_ formula) [41] immediately uses the *C*_T_ information, and the end result is normalized as a change in the expression of the target gene (IL6) in the target sample (RAS) compared to a shown reference sample (control) to a reference gene (GADPH). The mRNA expression levels of the IL6 gene were 1.88-fold changed in the RAS group, and there was a significant association between the expression of the IL6 gene and elevated risk of RAS (*p* < 0.001) (Table 2).

### 3.3. Methylation Status and mRNA Expression

**Figure 3:**
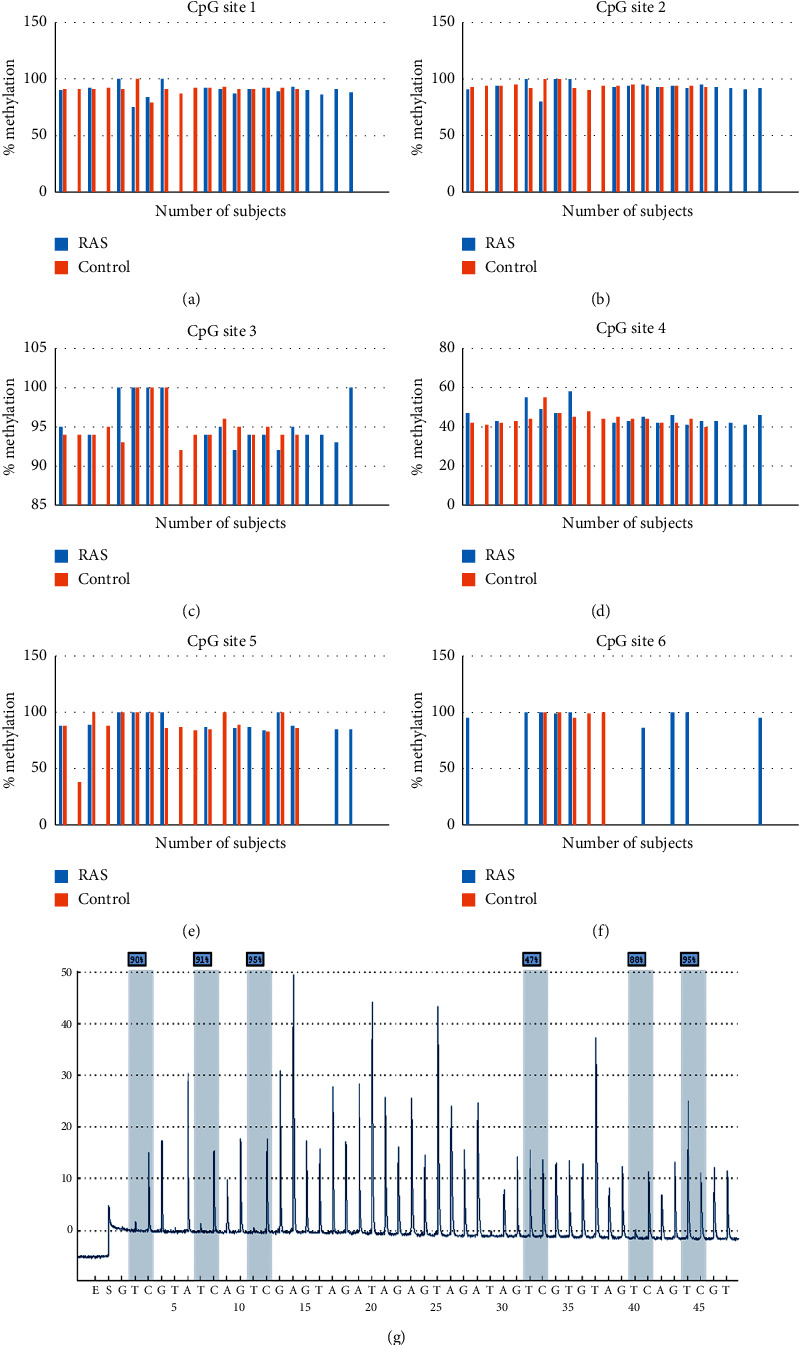
(a) Percentage of methylation in CpG site 1. (b) Percentage of methylation in CpG site 2. (c) Percentage of methylation in CpG site 3. (d) Percentage of methylation in CpG site 4. (e) Percentage of methylation in CpG site 5. (f) Percentage of methylation in CpG site 6. (g) Pyrosequencing of DNA Methylation in the 6 CpG island IL6 promoter of an RAS patient.

The six CpG sites of the IL6 gene in RAS and control groups were evaluated (Figure 3), and the probability of DNA methylation in the RAS group was equal to the control group (Table 3). The pyrosequencing result of the IL6 gene promoter region in an RAS patient (Figure 3(g)).

## 4. Discussion

The present study is the first study linking DNA methylation on IL6 expression, hematinic factors, and atopy in RAS subjects. Furthermore, it was reported that female subjects experience more RAS, although based on gender, it was not statistically significant between the two groups (*p*=0.305). Previous research has shown that females experience RAS more frequently than males [2, 46]. Women have a higher tendency to seek medical care of their disease or discomfort condition and be stressed higher than men [46]. The mean age in the RAS group was not significantly different from that of the control group (*p*=0.405). The age range of the subjects was selected according to epidemiological information showing the most common age of RAS at 20 [47]. Productive age tends to have physical and emotional stress, while it is closely related to increased serum cortisol level [48].

A statistically significant difference was observed in serum vitamin B-12 levels. In addition, the serum levels were reported in about 78% of the RAS subjects with levels above normal (hypercobalamin), and about 95% in the control group experience high levels (hypercobalamin). This indicates a functional and qualitative deficiency in vitamin B12, which is related to impaired absorption in the tissues and its effect. Furthermore, supplements or food containing vitamin B-12, such as liver, red meat, eggs, shellfish, or dairy products, potentially lead to hypercobalamine [49]. However, it was reported in RAS subjects according to the food frequency questionnaire that when eggs and milk are consumed almost daily, it can affect the emergence of hypercobalamin conditions, but further investigation is needed to ascertain the exact cause and to be aware of more severe disease.

The ferritin level did not differ between the two groups. This is in concordance with the results of the study conducted by Bao et al., and they also showed no significant difference in the decrease of ferritin and folic acid levels between the RAS and control groups [50]. Furthermore, the folic acid levels were normal in both groups, and the difference was not significant. These results were the same as in previous studies, which reported a minority of patients with deficiencies of folate associated with gastrointestinal disorders such as gastritis, peptic ulceration, and appendectomy, medications, HbE trait, no systemic diseases, or stress [51].

The IgE levels in the two groups did not differ significantly, possibly because the RAS subjects had no recurrence of the atopic disease episode during the study, or during the atopy therapy, in such a way that it affected the decrease in serum IgE levels. There was more history of atopic dermatitis than other atopic diseases. The IgE examination results were not high. Only six RAS subjects showed IgE > 200 IU/mL. Therefore, the number that can be categorized as atopy is only ten in the RAS group, and other subjects need further investigations. In addition, an increase in the IgE level in RAS patients was reported based on several previous studies related to a history of allergies in RAS subjects. Another factor was an increase in the number of mast cells in the tissues surrounding the lesion [51, 52]. The correlation analysis on the results of IgE with IL6 in the RAS group showed statistically significant results (*p*=0.011) similar to previous studies, which showed that IL6 levels increased in the bronchial asthma group because IL6 can increase mast cell proliferation [52, 53].

Many inflammatory mediators, including IL-1, IL-2, IL-17, and IL-18, have been investigated in RAS [17, 54, 55]. This study investigates IL6 as proinflammatory mediators that play an essential role in RAS and in hematinic deficiency and atopy. Its serum level depends on the expression of the IL6 gene, and its gene expression is influenced by epigenetic factors. One of the epigenetic factors that may be linked to hematinic deficiency and atopy is DNA methylation. The methylation of cytosines in CpG dinucleotides is an important regulator of gene expression in the human genome.

The study that analyzes DNA methylation on RAS subjects, notably those linking with the expression of proinflammatory genes, has not been performed previously. However, a study conducted by Lu et al. showed the role of one of the epigenetic mechanisms in the upregulation of LncRNA (long noncoding RNA) cancer susceptibility gene 2 (CASC2), IL6, and IL-18 in RAS [56]. This study showed that the percentage of methylation at four CpG site promoter IL6 was lower in the RAS than in the control group. The other two CpG sites indicated were a higher percentage than the control, but it was not statistically significant. The DNA methylation is a methyl group that is covalently added (-CH3) to base C (cytosine) in the 5′-CpG-3′-dinucleotide. The term CpG is a base C connected by a phosphate binds to the base guanine (G) in a DNA sequence [57]. The addition of methyl is carried out by DNA methyltransferase enzymes to genes in regulatory elements such as promoters, which can further suppress gene function (silencing) or trigger gene expression (enhancing) [58].

Subsequently, it was reported that DNA methylation on IL6 gene promoters is believed to increase IL6 expression. The effects of vitamin B-12 and ferritin deficiency reveal the role of nutrients in DNA methylation. Previous studies showed that some food components could change the status of DNA methylation, such as vitamin B as a coenzyme in metabolism “one carbon,” methyl-contributing nutrients, micronutrients that can modify “one-carbon” metabolism, and bioactive food compounds that can modify the activity of DNA methyltransferase [59]. The “one carbon” metabolism is an interconnected network of biochemical reactions; a “one-carbon” unit obtained from methyl-contributing nutrients is then transferred biochemically and molecularly to DNA synthesis [60]. A vitamin B-12 deficiency can cause hyperhomocysteinemia along with the “methylfolate trap.” This can limit the availability of methyl for homocysteine remethylation and DNA methylation [59]. A vitamin B-12 deficiency is associated with a statistically significant hypomethylation according to the studies conducted by Piyathilake et al. [61]. The DNA methylation can be influenced by a deficiency in vitamin B-12 and ferritin or atopic conditions. However, hypomethylation or hypermethylation was not statistically proven. Some gene expressions can be inhibited or enhanced because of hypomethylation or hypermethylation conditions in the 5′ UTR region of the DNA template. This study revealed that IL6 expression was significantly higher in subjects with hematinic deficiency and atopy-related RAS, but its high expression was not related to DNA methylation. This might occur because of other epigenetic factors such as some unique microRNAs (miRNAs) profile. A further study on other epigenetic mechanisms of IL6 gene expression in iron, folic acid, vitamin B-12 deficiency, and atopy-related RAS was proposed.

The present study showed that the upregulation of mRNA IL6 was correlated with RAS, hematinic deficiency, and atopy. The upregulation suggests that IL6 gene silencing can be a future novel therapy for RAS, especially in patients with hematinic deficiency and atopy. DNA methylation of CpG islands promoter IL6 in the present study is at sites of 566–658 based on the primer used. Thus, it would be necessary to conduct a further examination at sites 1–565 or 659–1684 at the CpG promoter IL6 location to confirm the status of DNA methylation in RAS. Also, an alternative strategy is needed to lower the IL6 expression by inhibiting the transcription with another epigenetic modality such as noncoding RNA or histone modification.

Subsequently, it was further discovered that IL6 might be a novel target of RAS treatment, especially those related to hematinic deficiency and atopy. The tocilizumab is needed as a direct anti-IL6 agent or silencing the IL6 gene expression through nanoparticle devices, such as the humanized recombinant monoclonal antibody of the IgG1_*k*_ subclass produced using recombinant DNA technology. In addition, environmental factors that contribute to the DNA methylation changes and IL6 upregulation should be reduced as a primary RAS prevention.

## 5. Conclusions

The present study showed that IL6 expression in RAS is mainly influenced by vitamin B-12 deficiency. It is believed that the influence of vitamin B-12 deficiency on IL6 expression is closely related to DNA methylation. However, the upregulation of IL6 mRNA levels was significantly higher in RAS patients. Furthermore, it was suggested that IL6 is an essential proinflammatory in RAS, which might also be influenced by other epigenetic mechanisms. We realized that this study has some limitations in subject recruitment. Therefore, the next proposed study of those linking DNA methylation CpG islands promoter IL6 at sites 1–565 and noncoding RNA with IL6 expression among RAS comorbid with atopy is absolutely essential.

## Figures and Tables

**Table 1:** Characteristics of subjects.

Characteristics	Group		*p* value
	RAS (*n* = 23)	Control (*n* = 21)	
Gender^#^			0.305^*∗*^
Male	10 (43.5%)	6 (28.6%)	
Female	13 (56.5%)	15 (71.4%)	

Age (year)	23.5 (4.2)	22.7 (3.8)	0.405^*∗∗*^

Hematinic deficiency##			
Vitamin B-12 (pg/mL)	1435.2 (464.6–4387.4)	2150 (852.2–3086)	0.001^*∗∗*^
Ferritin (ng/mL)	52.7 (3.02–174.97)	45.26 (2.53–140.13)	0.597^*∗∗*^
Folic acid (ng/mL)	19.4 (8.08–40.64)	20.45 (6.03–46.84)	0.264^*∗∗*^

Atopy			0.001^*∗*^
Dermatitis atopic (DA)	8	0	
DA and bronchial asthma	2	0	
Nonatopic	13	21	

Total IgE (IU/mL)##	155.7 (2.3–234.8)	167.32 (73.7–363.8)	0.540^*∗∗*^

^*∗*^Chi-square test. ^*∗∗*^Mann–Whitney Test. #Average (standard deviation). ##Median (range).

**Table 2:** Gene expression in the RAS and control group.

IL6 gene expression (change fold)	Groups		*p* value^*∗*^
	RAS^*∗∗*^ (*n* = 23)	Control^*∗∗*^ (*n* = 21)	
Average (SD)	1.88 (0.157)	1 (0.343)	<0.011

^*∗*^Mann–Whitney *U* test. ^*∗∗*^Calculated by the formula of gene expression = 2^−ΔΔ^*C*_T_ (Livak Method) [41]. SD = standard deviation.

**Table 3:** Methylation DNA in the RAS and control group.

DNA methylation (%) (S)	Groups		*p* value^*∗*^
	RAS^*∗∗*^ (*n* = 23)	Control^*∗∗*^ (*n* = 21)	
1. S1	90 (75–100)	91 (79–100)	0.579
2. S2	93.5 (80–100)	94 (90–100)	0.580
3. S3	95.7 (92–100)	95 (92–100)	0.838
4. S4	43 (41–58)	44 (40–55)	0.578
5. S5	91.4 (84–100)	88.4 (38–100)	0.822
6. S6	97.2 (86–100)	99 (95–100)	0.797

^*∗*^Mann–Whitney *U* test. ^*∗∗*^Median (Range), *S* = site of methylation (CpG site).

## Data Availability

All the data used to support the finding of this study are included in the manuscript.
